# A New α + β Ti-15Nb Alloy with Low Elastic Modulus: Characterization and In Vitro Evaluation on Osteogenic Phenotype

**DOI:** 10.3390/jfb14090452

**Published:** 2023-09-01

**Authors:** Tatiani Ayako Goto Donato, Karolyne dos Santos Jorge Sousa, Pedro Akira Bazaglia Kuroda, Carlos Roberto Grandini

**Affiliations:** 1 Laboratório de Anelasticidade e Biomateriais, UNESP—Universidade Estadual Paulista, Bauru 17033-360, SP, Brazil; tatianidonato@gmail.com (T.A.G.D.); kksousa12@gmail.com (K.d.S.J.S.); carlos.r.grandini@unesp.br (C.R.G.); 2 Institute of Biomaterials, Tribocorrosion and Nanomedicine—IBTN, Bauru 17033-360, SP, Brazil

**Keywords:** biomaterial, titanium alloys, cytotoxicity, osteogenic cells

## Abstract

This study aimed to produce Ti-15Nb alloy with a low elastic modulus, verify its biocompatibility, and determine whether the alloy indirectly influences cellular viability and morphology, as well as the development of the osteogenic phenotype in cells cultured for 2, 3, and 7 days derived from rat calvarias. Two heat treatments were performed to modify the mechanical properties of the alloy where the Ti-15Nb alloy was heated to 1000 °C followed by slow (−5 °C/min) (SC) and rapid cooling (RC). The results of structural and microstructural characterization (XRD and optical images) showed that the Ti-15Nb alloy was of the α + β type, with slow cooling promoting the formation of the α phase and rapid cooling the formation of the β phase, altering the values for the hardness and elastic modulus. Generally, a more significant amount of the α phase in the Ti-15Nb alloy increased the elastic modulus value but decreased the microhardness value. After the RC treatment, the results demonstrated that the Ti-15Nb alloy did not present cytotoxic effects on the osteogenic cells. In addition, we did not find variations in the cell quantity in the microscopy results that could suggest cell adhesion or proliferation modification.

## 1. Introduction

Commercially pure titanium (CP-Ti) and some titanium alloys, including Ti-6Al-4V, are the materials of choice for dental and orthopedic implants [1,2]. These materials have excellent ductility, good corrosion resistance, and high biocompatibility due to a TiO_2_ nanometric passivation film [3]. However, some properties of these materials, such as the elastic modulus, are still high compared to cortical bone [4]. This high elastic modulus may lead to potential stress in the surrounding residual bone, inducing resorption and detrimental bone remodeling [5]. In addition, several studies have shown that aluminum and vanadium, elements of the Ti-6Al-4V alloy, could cause long-term toxic effects on tissues [6]. It has been found that vanadium can cause allergic reactions in human tissues, and releasing aluminum ions into the bloodstream contributes to developing neurological diseases [7,8,9,10].

New Ti-based alloys without toxic elements are being developed to avoid health problems associated with aluminum and vanadium. Prominent alloys contain niobium, tantalum, zirconium, and molybdenum with no cytotoxic elements [2,6]. These elements are β-stabilizers that alter titanium’s microstructure and mechanical properties, representing a new class of Ti-based alloys free of Al and V, as well as exhibiting low values for the elastic modulus, making them quite attractive to use as a biomaterial for orthopedic applications [3,11].

Titanium is a transition metal with a hexagonal compact crystalline structure (α phase) at room temperature. This element undergoes an allotropic transformation around 883 °C to a body-centered cubic crystalline structure (β phase). This characteristic offers the possibility of obtaining alloys with different microstructures depending on the elements that stabilize the two phases [12]. At room temperature, niobium has a crystalline structure different from titanium. It is body-centered cubic [12]. Additionally, niobium is a non-toxic metal with high biocompatibility and does not cause allergic reactions in humans [13]. Brazil is the leading producer of niobium, with about 90% of the world’s resources and 95% of worldwide production [14]. Given this economic and strategic advantage, research on the processing and development of metals containing niobium is crucial.

Silva et al. [15] studied the effect of niobium as a substitutional solute on the mechanical properties of Ti-Nb alloys (5 and 10 wt% Nb). They observed a crystalline structure with the predominance of α phase and microhardness values showing a tendency to increase with the niobium concentration. It was observed that the Ti-10Nb alloy has a lower elastic modulus than CP-Ti. More recently, Luz et al. [16] investigated the effect of microstructure on nanotube growth using micro-arc oxidation (MAO) with the Ti-10Nb biphasic alloy (α + β) and observed that a lamellar oxide structure grew on rich β phase regions, forming a wall ~1.36 µm above the nanotubes of the α phase oxides. In another paper, plasma electrolytic oxidation (PEO) was employed by Luz et al. [17] to form oxide films on Ti-10Nb and Ti-20Nb alloys, aiming to favor the osseointegration process and enhance the tribo-mechanical behavior. It was observed that the oxide films formed on the binary Ti-Nb alloys were harder than their substrates, presenting lowered elastic modulus values in comparison to CP-Ti, and wear tests showed that the oxide layers of the electrochemically treated Ti-10Nb alloy were less damaged. Additionally, neither of the treated Ti-Nb alloys exhibited any sign of cytotoxicity in their oxide layers. 

Kuroda et al. [18] investigated as-cast and after-homogenization heat-treatment Ti-15Nb alloy for use as biomaterials, analyzing the effect of niobium addition on the phase stability, mechanical properties (such as hardness and elastic modulus), and cytotoxicity of the alloy. The alloy is biphasic and more rigid than CP-Ti, with a lower elastic modulus. No cytotoxic effects were observed, but other analyses should be undertaken to confirm if mutagenicity and other biological effects exist. 

Çaha et al. [19] studied the degradation behavior of Ti-15Nb and Ti-40Nb alloys in a simple physiological solution at body temperature in comparison with Ti-6Al-4V, the most popular Ti alloy, by performing corrosion tests during prolonged periods of immersion (up to 21 days), as well as by performing tribocorrosion tests against a hard and inert counter material. Although β-type Ti-40Nb alloy appeared to be promising for osseointegrated implants due to its lower elastic modulus (51 GPa), it exhibited lower corrosion resistance and lower resistance to tribocorrosion, mainly due to the elevated influence of delamination wear. 

**Figure 1:**
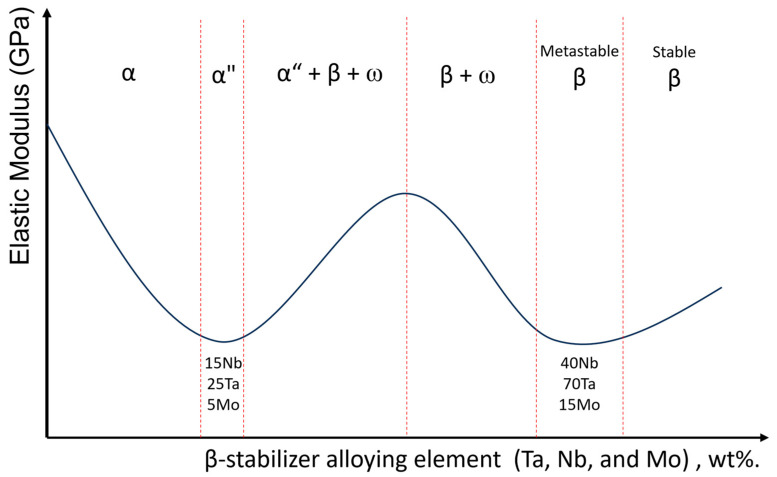
The titanium elastic modulus value changes due to the addition of β-stabilizing elements (Ta, Mo, and Nb).

In the production of new titanium alloys with low elastic moduli, the Ti-15Nb, Ti-40Nb, Ti-25Ta, Ti-50Ta, Ti-5Mo, and Ti10Mo alloys stand out. These alloys have low elastic modulus values due to the action of β-stabilizing elements (Ta, Nb, and Mo). Figure 1 illustrates titanium alloys’ elastic modulus value changes with the addition of isomorphic β-stabilizer elements [20,21,22]. It can be observed that commercially pure titanium (α-HCP) has a high elastic modulus. With the addition of small quantities of β-stabilizing elements, there is a drop in the value of the elastic modulus due to the decrease in the atomic bonding forces that substitutional atoms cause.

The elastic modulus value decreases to a limit value, and the α phase changes to the orthorhombic structure (α″); at this point, single-phase α″ alloys tend to have low values for the elastic modulus, a condition in which the Ti-15Nb alloy is found.

With the formation of the β and ω phases, there is an increase in the elastic modulus (generally, a small amount of Zr is used in an alloy to suppress the formation of the ω phase and decrease the intensity of the peaks that represent the increase in the elastic modulus in alloys with the chemical composition in this region of the diagram shown in Figure 1 [23]). With the suppression of the ω phase, there is again a decrease in the elastic modulus; at this point, the metastable single β-phase alloys will also have low elastic modulus values (Ti-15Mo, Ti-40Nb, and Ti-70Ta). Finally, adding more β-stabilizing elements to form an alloy with titanium increases the stability of the β phase, increasing the atomic binding energy and the elastic modulus.

Therefore, the Ti-15Nb alloys represent a new class of Ti-based alloys free of aluminum and vanadium while exhibiting a lower elasticity modulus than CP-Ti and Ti-6Al-4V alloys, which makes it quite attractive as a biomaterial.

A commonly used model for understanding numerous bone formation characteristics in vitro is the culture of osteogenic cells derived from rat tissue. These models allow an understanding of numerous characteristics, such as differentiation, cytokine and hormone regulation, synthesis, and the molecular mechanism of the formation of bone matrix [24,25]. For these bone-related characteristics to be expressed in vitro, the culture medium needs to be supplemented with mineralizing factors, such as ascorbic acid (AA) and β-glycerophosphate (βGP) [26,27,28,29].

Stepanovska et al. [30] analyzed the growth and osteogenic differentiation of mesenchymal stem cells derived from rat adipose tissue on the surface of commercial Ti-6Al-4V alloy with different surface roughness values. According to the authors, even though Al and V are considered cytotoxic, osteoblastic cells adhered to the Ti-6Al-4V alloy and proliferated well. However, a rough surface of approximately 60 nm improves adhesion and osteogenic differentiation.

This study aimed to produce the T-15Nb alloy and perform heat treatments to change the crystalline structures to optimize the elastic modulus value for biomedical purposes. It also verified the biocompatibility and biomineralization in vitro of the osteogenic cells on Ti-15Nb alloy. There are no reports in the literature on the biomineralization in vitro of osteogenic cells on Ti-15Nb alloy.

## 2. Materials and Methods

### 2.1. Sample Preparation

The Ti-15Nb alloy was obtained by melting the precursor materials in an arc furnace under an argon atmosphere to avoid contamination. For the melting process, the precursor materials (CP-Ti and pure Nb) were pickled using an acidic solution in a 4:1 volumetric ratio of nitric and hydrofluoric acid to remove surface impurities (gaseous and metallic). Due to the high reactivity of the element titanium with oxygen gas, the melting process must be carried out in a controlled atmosphere. Therefore, a mechanical vacuum pump (10^−2^ mbar) was used to remove the atmospheric gases inside the melting furnace. For the dielectric breakdown to occur in the production of the electric arc responsible for the melting of the precursor metals, it is necessary to insert inert argon gas (Ar) to be ionized, avoiding gaseous contamination. This procedure is a standard method for obtaining titanium alloys [31]. 

Metallic materials produced in arc melting furnaces are cooled in a copper crucible and kept close to room temperature through continuous cooling with running water. Due to this, there is a gradient in the cooling rate of the ingots, which can produce alloys with non-homogeneous crystalline structures (the base of the alloy in contact with the copper crucible cools faster). Therefore, heat treatments are performed to homogenize the crystalline structures of titanium alloys. The Ti-15Nb alloy was submitted to an annealing heat treatment (1273 K, 60 s, and 10^−6^ Pa vacuum) followed by slow cooling (SC) (−5 °C/min) and rapid cooling (RC) with ice water.

To verify the chemical composition, stoichiometry, and homogeneity of the Ti-15Nb alloy, composition analyses were carried out using energy-dispersive X-ray spectroscopy (EDS) in an Oxford INCA model.

The structure of the Ti-15Nb alloy (as-cast, SC, and RC conditions) was analyzed with X-ray diffraction measurements. X-ray diffraction measurements were performed with a Rigaku diffractometer (D/Max 2100PC) that emits copper Kα radiation (λ = 1544 Å). An Olympus BX51M microscope was used to analyze the alloys’ microstructure. Images were obtained at 200× and 1000× magnifications. 

To obtain micrographs showing the topography of the Ti-15Nb alloy under the influence of heat treatments, the Ti-15Nb alloy was sanded for 10 min with water sandpaper with granulometric measurements of 360, 400, 500, 600, 800, 1000, 1200, and 1500 mesh. Subsequently, the alloy’s surface was polished using a 1-micron alumina suspension to obtain a flat, mirrored surface without scratches. After the metallographic preparation, a chemical solution known as Kroll (16:3:1 of H_2_O, HN, and HF) was used to reveal the surface and microscopic structures, allowing the visualization of metallic grains.

To carry out the microhardness measurements, a Shimadzu HMV-2 microdurometer was used. Five indentations were made on the sample using 0.245 N force (applied load of 25 g) for 15 s [32]. Before carrying out the hardness tests, the Ti-15Nb alloy was again submitted to the metallographic preparation process.

A Sonelastic^®^ (Ribeirão Preto, Brazil) equipment was used for the elastic modulus measurements based on the impulse excitation technique. Each material emits a characteristic sound when struck that depends on its elastic properties, geometric dimension, and mass. The emitted sound contains information that allows the determination of elastic properties, such as the elastic modulus and the natural vibration frequencies [33].

### 2.2. Cell Culture

Osteogenic cells were isolated through sequential enzymatic digestion with 0.25% trypsin (Gibco-Invitrogen, Grand Island, NY, USA) and collagenase type II (Gibco-Invitrogen) of calvaria bone from new-born (2-day-old) Wistar rats, as previously described. All animal procedures were approved (Ethical Protocol #004/2016). Only isolated cells were grown in a complete α-minimum essential medium (α-MEM, Invitrogen, Carlsbad, CA, USA) supplemented with 10% fetal bovine serum (FBS) and 1% gentamicin (Invitrogen). The cells were incubated under standard cell culture conditions (37 °C, 95% humidity, and 5% CO_2_), and the medium was changed every two days [34].

### 2.3. Indirect Cytotoxicity Test

Ti-15Nb alloy disks (~6 mm in diameter and 2 mm thick) were cut using a diamond blade in a Buehler cutting machine (Isomet 100 model), cleaned by sonication, and autoclaved for the cytotoxicity tests. For the preparation of the extract, a complete medium was added at a concentration of 1 g/mL for 48 h under standard cell culture conditions (37 °C, 95% humidity, and 5% CO_2_).

A standard colorimetric assay using 3-(4,5-dimethyl-2-thiazolyl)-2,5-diphenayl-2H-tetrazolium bromide (MTT assay (Sigma, St. Louis, MO, USA)) was used to estimate cell viability and proliferation [35,36]. The indirect cell viability of the Ti-15Nb alloy was evaluated after heat treatment (RC) with the MTT assay at 2, 3, and 7 days [34]. The cells were cultured (density of 110 cells/mm²) on 13 mm polystyrene coverslips (Themanox^®^) for 2, 3, and 7 days, and the culture medium was substituted for the obtained extract (1 g of alloy/10 mL medium at 48 h). 

**Figure 2:**
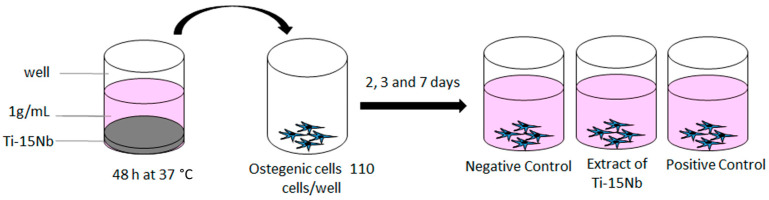
Schematic of the experiment setup for the analysis of biocompatibility of extracts of Ti-15Nb alloy.

The osteogenic cells were cultivated on 96-well microplates for an indirect cytotoxicity test. The polystyrene was used as the negative control (no cytotoxicity—base medium), while a solution of α-MEM, 10% FBS, and 1% phenol was used as a positive control (cytotoxicity). Figure 2 shows a schematic of the experiment setup for the analysis of biocompatibility. After the experiment, the samples were routinely processed for the MTT assay [35,37]. The wells’ optical density (OD) was determined using a plate reader at a test wavelength of 640 nm in a SpectraMax Plus microplate reader (Molecular Devices).

### 2.4. Scanning Electron Microscopy

The indirect cell morphology of the Ti-15Nb alloy was evaluated after heat treatment. The cells were cultivated under the surface of the glass, and the base medium was replaced by the extracts of the alloys (1 g/mL) for 2, 3, and 7 days. After this time, the cells were fixed in 4% glutaraldehyde (Electron Microscopy Sciences, Washington, PA, USA), rinsed with 0.2 M sodium cacodylate buffer (pH 7.4; Sigma-Aldrich), post-fixed with 1% osmium tetroxide (Sigma-Aldrich), and then rinsed in sodium cacodylate buffer. They were then routinely dehydrated by crescent alcohol concentrations followed by hexamethyldisiloxane (Electron Microscopy Sciences) for scanning electron microscopy (SEM) [29]. Samples were mounted onto aluminum substrates, sputtered with 20 nm gold, and examined in a Carl Zeiss EVO LS-15 model SEM. The base medium was used as the negative control.

### 2.5. Alizarin Red Staining

For the evaluation of calcium deposits, the cells were grown on glass coverslips (~13 mm), and the base medium was replaced by the Ti-15Nb alloy extract (1 g/mL) for seven days. A base medium (α-MEM + 10% FBS + 1% gentamicin) was used as a negative control with mineralization. The base medium supplemented with 50 µg/mL AA (Sigma, St. Louis, MO, USA) and 10 mM βGP (Sigma) was used for the positive control. After these periods of treatment, the cells were rinsed with Hanks’ balanced salt solution (Sigma), fixed with 70% ethanol for 1 h at 4 °C, and stained with 2% alizarin red S (Sigma), pH 4.2, for 15 min at room temperature (RT) [38].

## 3. Results and Discussion

**Figure 3:**
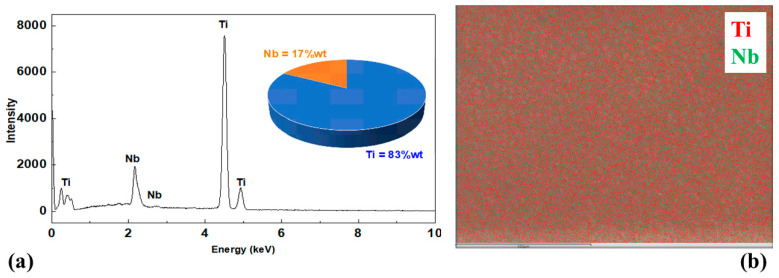
Energy-dispersive X-ray spectroscopy: chemical composition (**a**) and titanium and niobium elements (**b**) mapped for Ti-15Nb alloy after melting.

Figure 3 presents the results of the quantitative analysis using EDS of the elements that composed the Ti-15Nb alloy. The chemical analysis (Figure 3a) showed that the alloy’s base elements remained close to the nominal values. Figure 3b shows the mapping of the elements present in the sample. The red points represent titanium, and the green points represent niobium. It is impossible to observe any segregates or precipitates in the image, which shows a good distribution, indicating that the produced alloy was of good quality.

**Figure 4:**
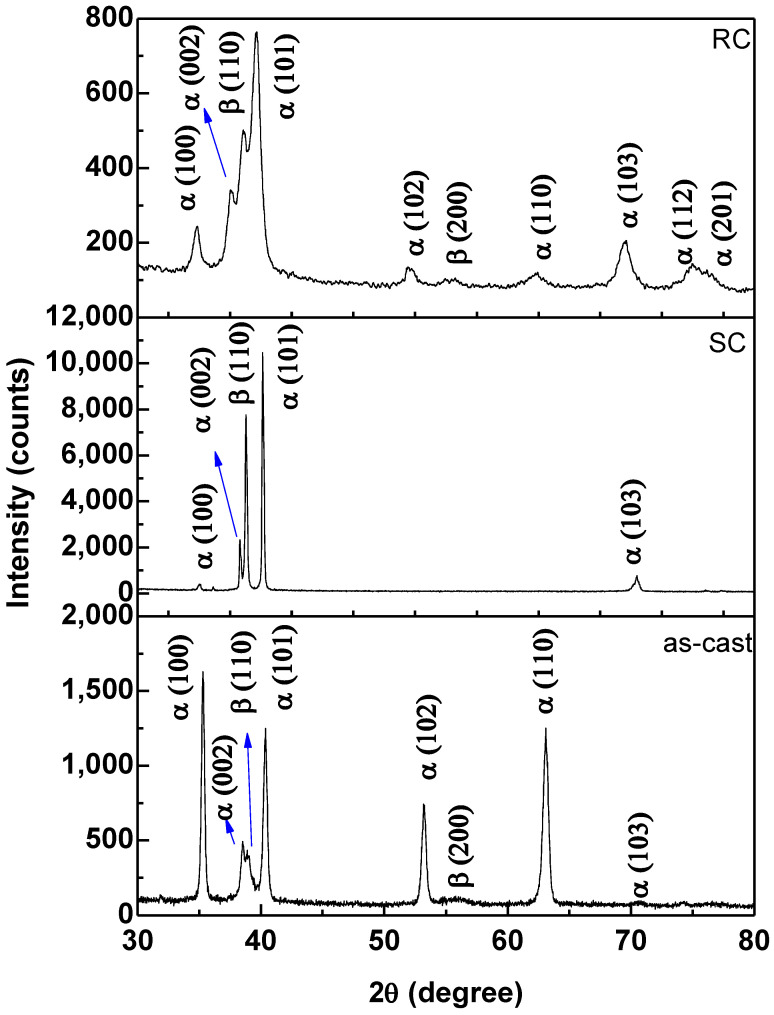
XDR patterns from Ti-15Nb sample after melting (as-cast) and heat treatments with slow (SC) and rapid cooling (RC).

Figure 4 shows the structural characterization results obtained with the X-ray diffraction technique. From the diffractograms, it can be seen that the Ti-15Nb alloy was of the α + β type; that is, it had two crystalline structures: compact hexagonal (α) and body-centered cubic (β). These two crystalline phases belong to the stable phases of titanium and are obtained at different temperatures. α-hcp is stable at temperatures below 862 °C; on the other hand, titanium has the β-CCC structure above this temperature. In this sense, it can be observed that the addition of the Nb element decreased the allotropic transformation temperature because it is a β-stabilizing element [18].

For the as-cast condition, it can be observed that the α peaks (100), (102), and (110) were more intense than the prominent peak (101), showing that there was a preferential orientation of the crystallographic planes. The emergence of these planes was due to the nonuniform cooling rate of the ingot in contact with the water-cooled copper crucible. Heat treatments followed by slow and rapid cooling (SC and RC) efficiently eliminated this type of texture and preferential orientation, and the α phase peak (101) became the most intense, as expected for HCP alloys without the influence of textures and deformations.

In the Ti-15Nb alloy subjected to heat treatment followed by slow cooling (SC), it can be observed that the presented diffractogram has high intensities, and the peaks are thinner. This behavior was due to the cooling conditions of the Ti-15Nb alloy because, when heated above the β-transus temperature (1000 °C), the crystalline structure completely changes to β; following slow cooling (−5 °C/min), the Ti and Nb atoms rearranged and ordered themselves in their lowest energy state, showing stable α and β phases. 

Regarding the heat treatment followed by rapid cooling (RC), there was a decrease in the intensity and widening of the peaks; in addition, β peaks were more widely present in this condition, indicating that rapid cooling with water was able to retain a more significant amount of β phase. In this sense, the Ti-15Nb alloy in the RC condition can be expected to have a lower elastic modulus because it has more β phase.

**Figure 5:**
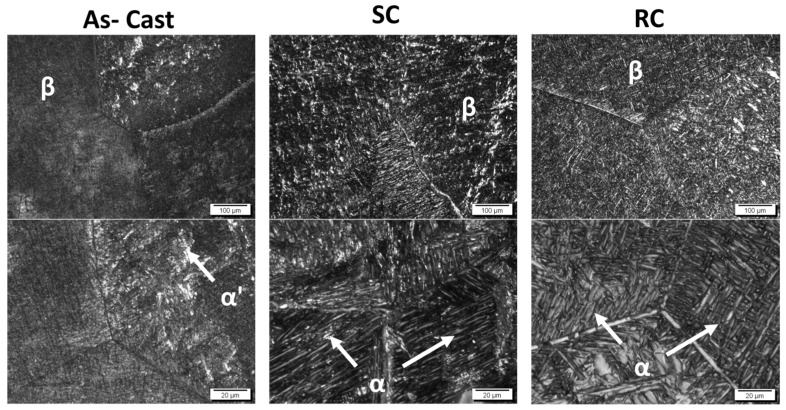
Ti-15Nb alloy micrographs after melting (as-cast) and heat treatments with slow (SC) and rapid cooling (RC).

Figure 5 shows micrographs of the Ti-15Nb alloy (as-cast, SC, and RC) to corroborate the XRD results. In all conditions studied, an acicular structure characteristic of α-type titanium alloys could be visualized in the intra-grain area of the β matrix. In the Ti-15Nb alloy subjected to heat treatment followed by slow cooling (SC), the α acicular structures were more straightforward to visualize due to a more significant formation of the alpha phase since the slow cooling in the α + β type alloys tends to promote the formation of the α-HCP phase.

**Figure 6:**
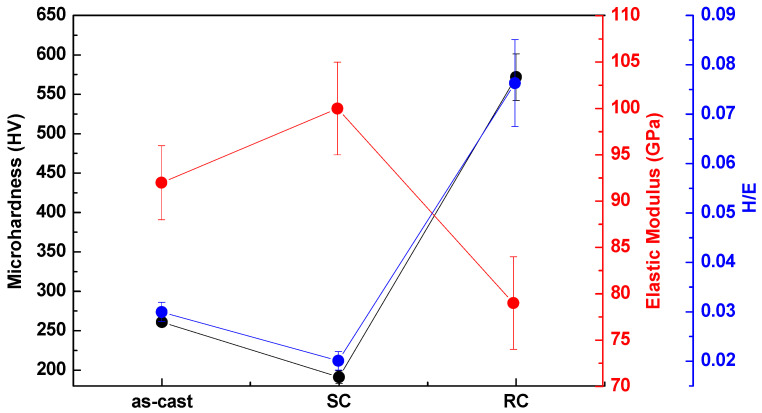
Microhardness (black line), elastic modulus (red line), and H/E ratio (blue line) values obtained from the Ti-15Nb alloy.

The data obtained regarding the microhardness, elastic modulus, and H/E ratio of Ti-15Nb alloy (as-cast, SC, RC) are shown in Figure 6. It can be observed that Ti-15Nb alloy was more rigid than CP-Ti, Ti-5Nb, and Ti-10Nb alloys [15]. In the Ti-15Nb alloy subjected to heat treatment followed by slow cooling (SC), no difference was observed compared to the Ti-10Nb alloy. As shown, the alloys have hardness values significantly higher than that of CP-Ti, indicating that this high mechanical resistance results from hardening via the solid solution caused by adding niobium [39]. It can also be observed that the SC heat treatment, used to remove the internal stresses arising from casting, decreased the microhardness values [11]. In the RC condition, there was alloy hardening due to rapid alloy cooling promoting the β phase.

Lee et al. have explained the change in hardness of titanium alloys of the Ti-Nb system under the influence of the crystalline structure [40]. For these authors, the ω phase had the highest hardness value among all the crystalline phases of titanium, followed by the α′, α″, β, and α phases. Guided by the trend presented by Lee et al., the decrease in the hardness value in the heat treatment followed by slow cooling (SC) condition and the increase in the hardness value in the rapid cooling (RC) condition can be explained. The Ti-15Nb alloy after heat treatment with slow cooling (SC) had a high amount of α phase (slow cooling promotes the formation of the α phase), so it had a lower hardness value, whereas the alloy in the RC condition had a more significant amount of β; as a consequence, it had a higher hardness value.

Concerning the elastic modulus, it is understood that titanium alloys with a higher amount of β phase tend to have a lower value for the elastic modulus. Thus, after heat treatment with rapid cooling (RC), the alloy had a lower elastic modulus (79 GPa) because it had a more significant amount of the β phase. On the other hand, α alloys tend to have a high modulus value (e.g., CP-Ti has approximately 100 GPa [41]), so the heat-treated alloy with slow cooling (SC) had a higher amount of α (100 GPa).

The H/E ratio, the quotient of the hardness value and the elastic modulus, is a simple analysis used to predict the wear resistance of titanium alloys [40]. The higher the H/E ratio is, the greater the probability that the alloy has good resistance to wear phenomena [42]. It can be observed that the alloy in the RC condition had a higher H/E value (~0.075), indicating that this study condition made the Ti-15Nb alloy more resistant to wear; in addition, it can be observed that the trend of the H/E curve followed the microhardness value, and, generally, metals with high hardness values tend to be resistant to abrasive wear.

**Figure 7:**
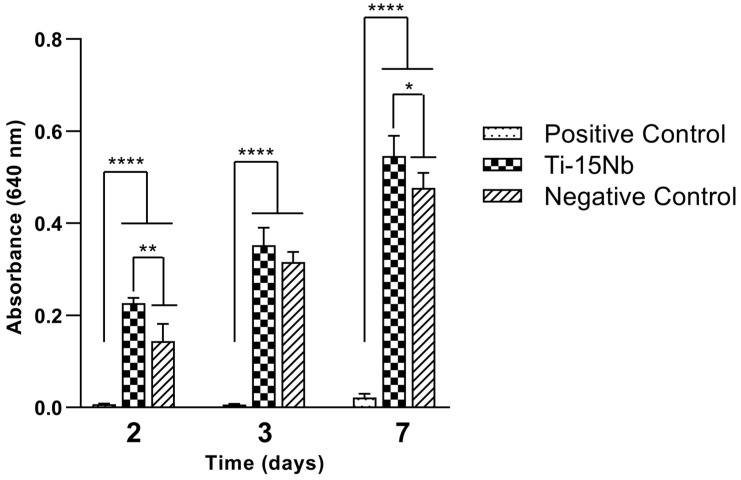
Indirect cytotoxicity test of Ti-15Nb alloy after RC heat treatment in a primary culture of osteogenic cells for 2, 3, and 7 days. PC: positive control—1% phenol; NC: negative control—polystyrene. ANOVA and post hoc Tukey. * *p* < 0.05, ** *p* < 0.01, and **** *p* < 0.001.

Figure 7 presents the indirect cytotoxicity test results for Ti-15Nb alloy after heat treatment followed by rapid cooling (RC) in a primary culture of osteogenic cells for 2, 3, and 7 days compared with 1% phenol, used as positive control, and polystyrene, used as the negative control. It can be observed that, for each day, the number of viable cells was similar to the negative control (no cytotoxicity), with no significant differences present. However, in all studied periods, the viable cells in the positive control (cytotoxicity—phenol solution) were significantly lower than in the extracts of the Ti-15Nb alloy. These results imply that the Ti-15Nb alloy did not release any cytotoxic elements that interfered with cell proliferation during the studied periods. However, this finding should not imply that studies with longer durations should be discarded [35,37].

**Figure 8:**
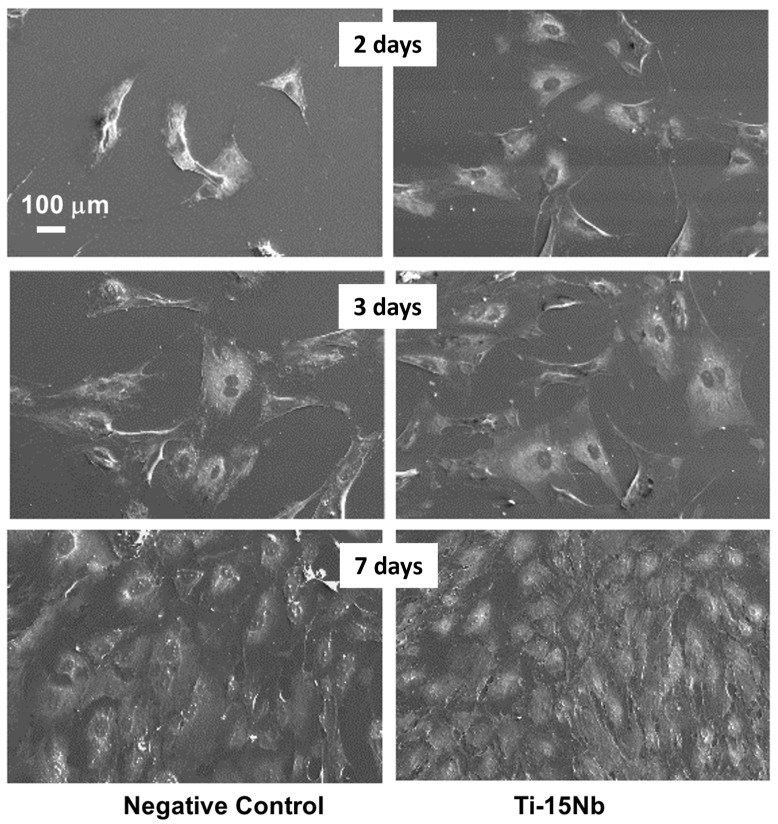
Indirect SEM analysis of Ti-15Nb alloy after RC heat treatment in a primary culture of osteogenic cells for 2, 3, and 7 days. The base medium was used as the negative control.

The SEM micrographs from the indirect cytotoxicity test for the Ti-15Nb alloy after heat treatment with rapid colling (RC) in a primary culture of osteogenic cells for 2, 3, and 7 days compared with the base medium used as the negative control are presented in Figure 8. The SEM results showed that the cell morphology of the extracts of Ti-15Nb alloy after RC heat treatment was similar to the negative control. Good adherence with a central and flattened cell body and numerous long cellular processes were observed in the Ti-15Nb and negative control [43]. Previous studies have shown evidence that titanium alloys containing elements such as zirconium, niobium, and tantalum are non-toxic in cytotoxicity investigations and preserve the original cell morphology with triangle, polygon, and fusiform shapes, as well as cytoplasmic extensions, which are characteristic elements of this primary culture [18,43,44,45].

**Figure 9:**
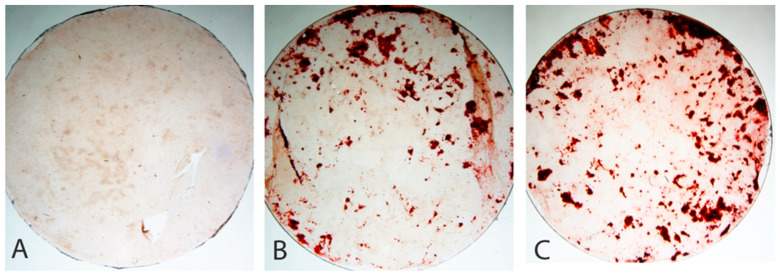
Indirect alizarin red analysis of Ti-15Nb alloy after RC heat treatment in a primary culture of osteogenic cells for seven days. (**A**) Negative control with mineralization (base medium); (**B**) extract of Ti-15Nb alloy supplemented with AA + βGP; (**C**) positive control with mineralization (base medium supplemented with AA + βGP).

Figure 9 presents an indirect alizarin red analysis for Ti-15Nb alloy after RC heat treatment in a primary culture of osteogenic cells for seven days. The presence of mineralized nodule formation, evaluated by alizarin red staining, supported the cell viability assay. In the cells without supplementation with AA + βGP (base medium), no mineralized bone-like nodule formation was identified after seven days, as can be seen in Figure 9A. On the other hand, a significant increase in nodular staining was observed in the cell culture with the Ti-15Nb alloy extract after the RC heat treatment supplemented with AA + βGP (Figure 9B), as well as in the positive control group (AA + βGP), as shown in Figure 9C. Similar data have been presented in recent studies with CP-Ti, where contact with this material did not interfere in the formation of a mineralized bone matrix, even without surface treatment [28,46,47,48].

These results suggest that applying RC heat treatment to the extract of the Ti-15Nb alloy did not alter the cell viability in all studied periods. The presence of viable cells was similar to the negative control. The cells exhibited good adhesion to the substrate, and the cell morphology was preserved. In addition, mineralized bone-like nodule formation was identified in the cells treated with this extract for an extended period, and the positive control with mineralization prevented the differentiation of calvaria-derived osteogenic cells into osteoblasts [18,28,46,47,48].

## 4. Conclusions

The structural and microstructure characterization techniques allowed us to conclude that the Ti-15Nb alloy is an α + β-type titanium alloy.

Heat treatments carried out above the allotropic transformation temperature (1000 °C) followed by slow cooling (SC) promoted α phase formation; on the other hand, rapid cooling (RC) promoted the β phase.

The β and α phase amounts influenced the values for the elastic modulus and hardness of the Ti-15Nb alloy. The Ti-15Nb alloy with a higher α phase had a high modulus value (100 GPa) and low hardness value (200 HV); in contrast, the Ti-15Nb alloy rich in β phase had a high hardness value (550 HV) and a low elastic modulus (78 GPa).

In accordance with the H/E ratio, the alloy with the RC condition tended to have a higher wear resistance (~0.07) due to its high hardness.

The biocompatibility and the biomineralization in vitro of the osteogenic cells on Ti-15Nb alloy after the RC heat treatment were analyzed.

The extract of Ti-15Nb alloy did not affect the cell viability and morphology, inducing the differentiation of calvaria-derived cells into osteoblasts and allowing the development of the osteogenic phenotype in indirect contact with this alloy.

The Ti-15Nb alloy is an excellent candidate to use as a biomaterial for orthopedic applications because of its good mechanical properties and lack of cytotoxicity in the presence of osteoblastic cells.

## Data Availability

The data presented in this study are available on request from the corresponding author.
